# Recent advances on the development and regulation of flower color in ornamental plants

**DOI:** 10.3389/fpls.2015.00261

**Published:** 2015-04-27

**Authors:** Daqiu Zhao, Jun Tao

**Affiliations:** Key Laboratory of Crop Genetics and Physiology of Jiangsu Province, College of Horticulture and Plant Protection, Yangzhou UniversityYangzhou, China

**Keywords:** flavonoids, anthocyanidins, ornamental plants, physical and chemical factors, genetic engineering

## Abstract

Flower color is one of the most important features of ornamental plants. Its development and regulation are influenced by many internal and external factors. Therefore, understanding the mechanism of color development and its regulation provides an important theoretical basis and premise for the cultivation and improvement of new color varieties of ornamental plants. This paper outlines the functions of petal tissue structure, as well as the distribution and type of pigments, especially anthocyanins, in color development. The progress of research on flower color regulation with a focus on physical factors, chemical factors, and genetic engineering is introduced. The shortcomings of flower color research and the potential directions for future development are explored to provide a broad background for flower color improvements in ornamental plants.

## Introduction

Flower color can attract pollinators and protect floral organs. Furthermore, people enjoy these colors in daily life. For ornamental plants, flower color is an important quality determinant that not only affects the ornamental merit of a plant but also directly influences its commercial value. Although there is a wide range of natural flower colors, colors are limited in some important ornamental plants. For example, Chinese rose and chrysanthemum lack blue, and herbaceous peony and cyclamen lack yellow. Therefore, making flower color improvements has always been an important goal for breeders.

Over the years, much research has been conducted on the development and regulation of ornamental plant color. Researchers have found that the development of flower color is related to petal tissue structure, pigment distribution and its types; it can be regulated through environmental factors and genetic engineering. In this review, we described recent advances toward a better understanding of the development and regulation of flower color in ornamental plants.

## Mechanism of Flower Color Development

When a petal is exposed to light, the light penetrates the pigment layer and is partially absorbed. Some of the remaining light is reflected by the sponge tissue and passes back through the pigment layer. Therefore, it is sensed by our eyes as color. The color of flowers is related to the internal or surface tissue structure of a petal and the type and amount of pigments in the petal cells, but pigment plays a major role.

## Petal Tissue Structure and Pigment Distribution

**FIGURE 1:**
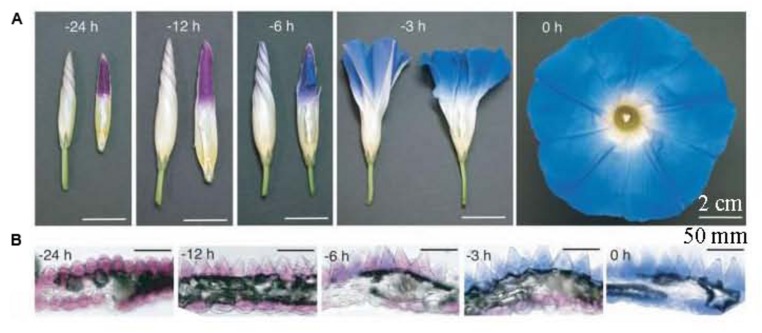
**Petal color change and transverse sections in the open process of Ipomoea tricolor (Yoshida et al., 2009). (A)** Whole flower growth. The right photos are half-cut buds; **(B)** Transverse sections of petals.

Petal tissue structure is similar to leaf blade structure, which can be divided into four parts: upper epidermis, palisade tissue, sponge tissue, and lower epidermis. Under normal circumstances, petal pigments are mainly distributed in the upper epidermal cells, but they can also be found in the palisade tissue and the lower epidermis of dark colored petals. For example, pigment exists in the palisade tissue of pale blue grape hyacinth (Qi et al., 2013) as well as in the petal lower epidermis of tulip (Shoji et al., 2007), Ipomoea tricolor (**Figure 1**; Yoshida et al., 2009) and meconopsis (Yoshida et al., 2006). Typically, no pigment is distributed in the sponge tissue. However, its thickness and density is related to the brightness of flower color. The thicker and denser the sponge tissue, the brighter the color (An, 1989).

Different pigments in the same tissue can exhibit different subcellular localization. In general, carotenoids are deposited in the plastids of cytoplasm, and flavonoids are deposited in vacuoles. It has also been found that flavonoids can exist in different forms in cells, Markham et al. (2000) reported the presence of flavonoids in lisianthus petal epidermal cell walls.

**FIGURE 2:**
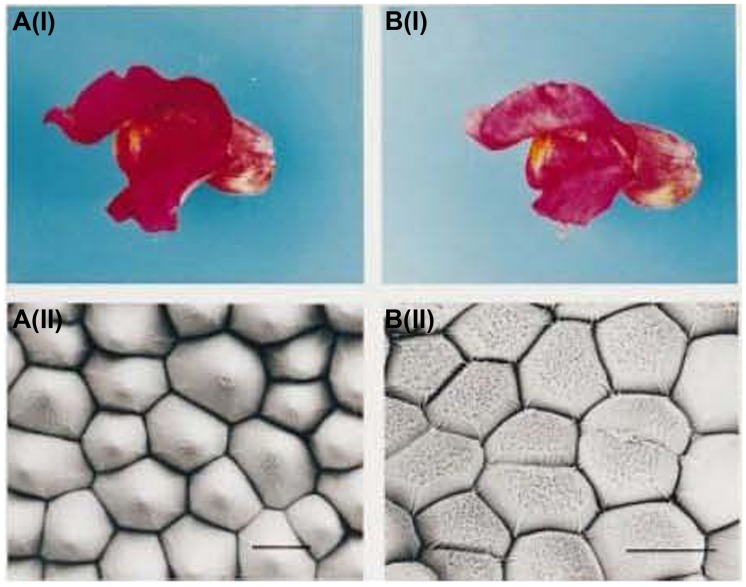
**Petal color and scanning electron micrograph of snapdragon and its mutant (Noda et al., 1994). A(i)** Wild-type flower with magenta petals; **A(ii)** Scanning electron micrograph of wild-type petal; **B(i)** Mutant flower with pink petals; **B(ii)**; Scanning electron micrograph of a mutant petal.

In addition, various shapes of petal epidermal cells can also have an important impact on flower color. Conical cells can increase the proportion of the incident light on epithelial cells, which enhances the light absorption by pigments, thereby leading to darkened flower color and enhanced color saturation. Flat cells can reflect more incident light, leading to lighter flower color. The epidermal cells with protruding papillae can generate a velvet sheen on the petals. Noda et al. (1994) found that when magenta snapdragon was mutated to pink, conical epidermal cells became flat (**Figure 2**), this transformation was regulated by a MYB family transcription factor, MIXTA. And Vignolini et al. (2015) found that the diffraction from the regularly folded cuticle overlying the petal epidermal cells in *Hibiscus trionum* generated the iridescent effect. In addition, Yoshida et al. (1995) believed that the length and arrangement of iris petal epidermal cells had certain influences on the flower color.

## Pigment Types

People have extracted pigments from colorful flowers to study their components since the mid-19th century. After more than 150 years of research, a wide variety of pigments have been found which could be generally divided into three groups, carotenoids, flavonoids, and alkaloids according to their chemical structures, cellular localizations and biochemical synthesis pathways.

Carotenoids are the most widely distributed pigments in nature. In addition to flowers, they can also be found in fruits, leaves and roots in higher plants. Carotenoids can be divided into the two major categories of carotene and lutein. Both groups are cyclization-produced organic molecules of a C40 polyene backbone with an ionone ring at the end. This structure makes carotenoids able to absorb visible light of short wavelengths. The wavelength of light being absorbed is determined by the number and properties of double bonds. Therefore, carotenoids can be brilliant red, orange and yellow (Britton et al., 2004). Although carotenoids exist in the petals of different ornamental species, their specific compositions are not the same in all species. Han et al. (2014) found that *Osmanthus fragrans* yellow petals contained small amounts of β-carotene, golden yellow petals had high levels of lutein, as well as low levels of α-carotene and β-carotene, and orange–red petals accumulated considerable concentrations of α-carotene and β-carotene. Previous studies have shown that the petals of marigold ‘Lady’ and chrysanthemum ‘Yellow Paragon’ contain only lutein (Moehs et al., 2001; Ohmiya et al., 2006). Large amounts of violaxanthin and zeaxanthin, as well as small amounts of neoxanthin, lutein, zeaxanthin and β-carotene, are the carotenoid components that make lotus root yellow (Suzuki et al., 2007). The major carotenoid components in yellow oncidium petals are trans-violaxanthin and 9-*cis*-violaxanthin (Hieber et al., 2006), whereas zeaxanthin, β-carotene and ζ-carotene are mainly found in saffron petals (Castillo et al., 2005). Other zeaxanthins, 9-Z-violaxanthin and *cis*-lutein are the main components of the yellow lily ‘Connecticut King’ petals (Zhu et al., 2010).

Flavonoids are a large class of secondary metabolites, which are widely distributed in plants. Chemically, flavonoids are a collection of substances based on the structure of the 2-phenylchromone nucleus. Flavonoids are the most important pigment group and produce the widest spectrum of colors, ranging from pale yellow to blue-purple. They are one of the most important pigments in a variety of ornamental plant petals, such as chrysanthemum (Chen, 2012), dahlia (Thill et al., 2012), groundcover rose (Schmitzer et al., 2010), violet (Fumi et al., 2012), and herbaceous peony (Zhao et al., 2012a,b, 2013, 2014). The composition of flavonoids may vary greatly among different color petals of the same species. Chen et al. (2012) analyzed the pigment composition of chrysanthemum in two different color flowers and found that the white flower contained only flavones and flavonols, whereas the pink flowers mainly contained anthocyanins, flavones and flavonols. He et al. (2011) analyzed the pigments of purple, red, orange, yellow, and white *Lycoris longituba* and found that only one of the four identified anthocyanins was present in all purple, red, and orange samples; no anthocyanins were detected within white and yellow samples. This result occurs mainly because among flavonoids, anthocyanin belongs to the red series and controls pink to blue-violet flower colors. Other flavonoids belong to the pure yellow series, among which chalcone and aurone are deep yellow, and flavones, flavonols and flavanones are light yellow or nearly colorless.

Alkaloids are a class of cyclic organic substances that contain negative oxidized nitrogen atoms, including betalain, papaverine and berberine. Among them, betalain is a water-soluble nitrogen compound present in red beets (also known as purple beetroots) and some flowers, fruits, roots and leaves. Betacyanin and betaxanthin are present in these plants, with betacyanin being the main component, accounting for ∼75–95% of the total betalain (Strack et al., 2003). To date, betalain has been found only in Caryophyllales plants (except Caryophyllaceae and Molluginaceae whose colors are produced by anthocyanin). The two types of pigments, betalains and anthocyanins, have never been found in the same plant (Gandía-Herrero and García-Carmona, 2013). Betalains are very important for flower color development. The difference between a flower being red or yellow depends on the presence of betacyanin or betaxanthin in the petals. Orange to red or variegated colors may be produced if both pigments co-exist in a flower (Gandía-Herrero et al., 2005; Felker et al., 2008). Kugler et al. (2007) researched amaranth and bougainvillea in three different colors, and found that the orange petals contained mostly betaxanthin and a minor amount of betacyanin; the red petals contained essentially equal amounts of the two pigments. A large amount of betacyanin was accompanied by a trace amount of betaxanthin in the purple petals.

## Anthocyanins and Color Development

Among of the aforementioned pigments, water soluble flavonoids containing anthocyanins and anthoxanthins can produce the full spectrum of colors from pale yellow to blue–purple. Anthoxanthins mainly produce the colors from white to dark yellow in flowers. And anthocyanins are the main flavonoid group, they play an irreplaceable role in the color development of plants, exhibiting a wide range of colors, from pink to blue–purple. Therefore, this section will review the role of anthocyanins in flower color development.

**FIGURE 3:**
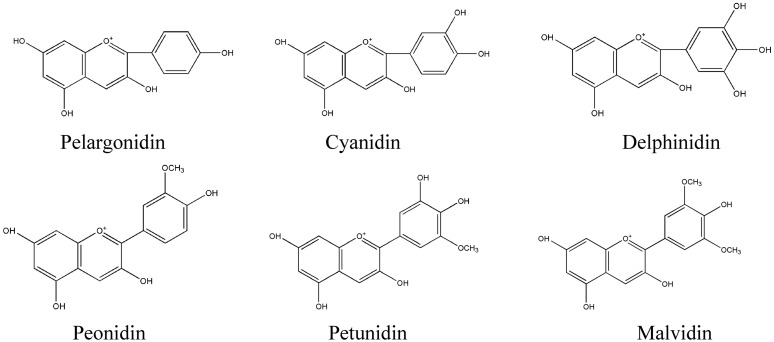
**Most important natural anthocyanidins in plants**.

As flavonoids, anthocyanidins have a highly characteristic C6-C3-C6 carbon skeleton and the same biosynthetic origins. Due to the instability of anthocyanidins, they exist mainly as anthocyanins (i.e., sugar-containing counterparts) in plants. Approximately 100 anthocyanins have been reported (Veitch and Grayer, 2008), primarily derived from six common types of anthocyanidins, namely, pelargonidin, cyanidin, delphinidin, peonidin, petunidin, and malvidin (**Figure 3**). In terms of biosynthesis, peonidin is derived from cyanidin, and petunidin and malvidin are derived from delphinidin; thus, pelargonidin, cyanidin, and delphinidin are the three main anthocyanidins (Tanaka et al., 2009). The anthocyanin sugar groups mainly include glucose, rhamnose, xylose, galactose and arabinose, and the monosaccharaides compose uniform or non-uniform disaccharides and trisaccharides; 3-monoglucoside, 5-diglucoside, 3,5-diglycoside and 3,7-diglycoside are the most common (Liu, 1998). The colors of the different anthocyanins are related to the environment and the substituents linked to the parental C6-C3-C6 carbon backbone.

Anthocyanins are a class of pigments that are soluble in water, methanol, ethanol, and acetone; they are insoluble in ether and chloroform. They can be precipitated by lead acetate and absorbed by activated carbon. Anthocyanin extract is distinguished from other flavonoids by strong visible light absorption. It exhibits a significant characteristic absorption peak at 500–550 nm in the visible region (Zhao et al., 2012b). Anthocyanins are very unstable. Light, temperature, pH, oxidants and reducing agents can significantly affect their stability (Bordignon-Luiz et al., 2007; Zhao et al., 2011). For example, the color of anthocyanins is red in acidic pH, colorless in neutral or nearly neutral pH and blue in alkaline pH. This effect is due to the existence of four anthocyanin tautomers in different pH values: alkali blue quinone A, red–yellow molten cation AH^+^, colorless false base B and colorless chalcone C. The three balance conversions between them are readily affected by pH (Pina, 2014).

Anthocyanins are glycosides, which are naturally formed by anthocyanidins and various sugars. They are stably localized in plant organs, such as petals, and are red, purple, blue, and black (Li et al., 2003). Previous studies have shown that the color differences are related to the anthocyanin content. Kazuma et al. (2003) measured the amount of anthocyanins in a series of butterfly pea petals from white to blue and found that the anthocyanin content was significantly higher in the blue petals than in the other petals; there were no anthocyanins in the white petals. The differences in anthocyanins are one of the important reasons for the development of a variety of colors. In cineraria, the blue and red flower colors are mainly determined by delphinidin aglycone and cyanidin aglycone, respectively. The pink flowers contain cyanidin aglycone and pelargonidin aglycone as the core anthocyanins, and purple flowers contain mainly delphinidin aglycone and cyanidin aglycone as the core anthocyanins (Sun et al., 2009). The red pigments, pelargonidin and cyanidin, appear differently in lagenaria. Cyanidin appears in red, while pelargonidin leans toward scarlet (Zhang et al., 2011). Moreover, the glycoside types of the same anthocyanidins are also closely linked to flower color development. In tropical water lily, the cultivars which are detected delphinidin 3-galactoside (Dp3Ga) present amaranth, and detected delphinidin 3′-galactoside (Dp3′Ga) present blue (Zhu et al., 2012). In addition, the co-coloring effect, the pH in the vacuole and chelation are all important in affecting the color of anthocyanins, which have been described in detail by Tanaka et al. (2009).

## Anthocyanin Biosynthetic Pathway and Key Genes

**FIGURE 4:**
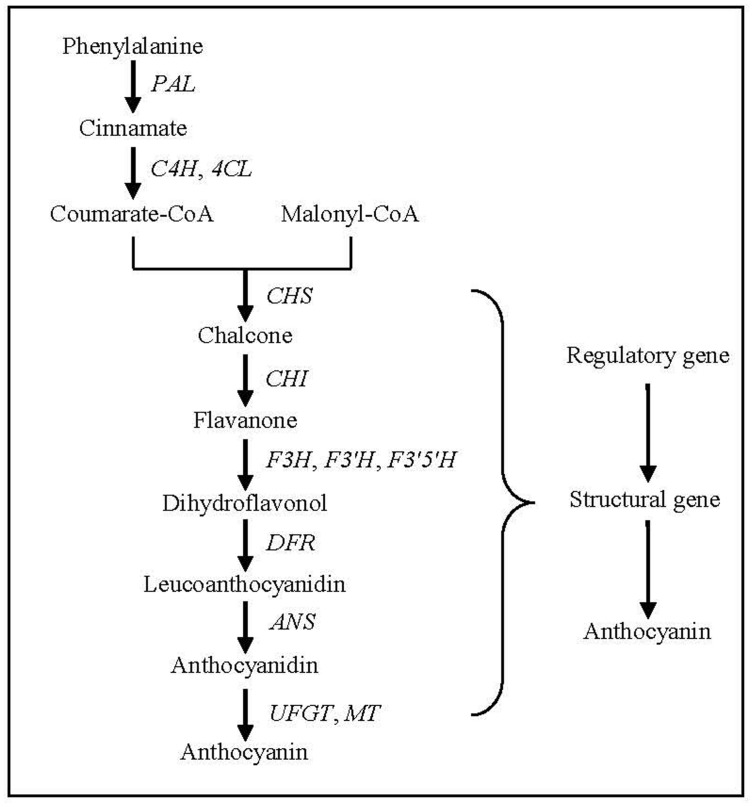
**Anthocyanin biosynthesis pathway in plants**. PAL, phenylalanine ammonia lyase gene; C4H, cinnamate-4-hydroxylase gene; 4CL, 4-coumarate: CoA ligase gene; CHS, chalcone synthase gene; CHI, chalcone isomerase gene; F3H, flavanone 3-hydroxylase gene; F3′H, flavonoid 3′-hydroxylase gene; F3′,5′H. flavonoid 3′,5′-hydroxylase gene; DFR, dihydroflavonol 4-reductase gene; ANS, anthocyanidin synthase gene; UFGT, UDP-glucose: flavonoid glucosyltransferase gene; MT, methyl transferase gene.

Anthocyanin biosynthesis has been a research hotspot in the field of plant secondary metabolism, and its biosynthetic pathway and key genes in plants have been clarified (Cheynier et al., 2013). Anthocyanin biosynthesis, beginning with the direct precursor of phenylalanine, can be divided into three stages (**Figure 4**). The first stage is the conversion of phenylalanine to coumarate-CoA by phenylalanine ammonia lyase (PAL), cinnamate-4-hydroxylase (C4H) and 4-coumarate: CoA ligase (4CL), which is a common step in many secondary metabolic pathways. The second stage is the formation of dihydroflavonol by one molecule of coumarate-CoA and three molecules of malonyl-CoA catalyzed by chalcone synthase (CHS), chalcone isomerase (CHI), flavanone-3-hydroxylase (F3H), flavonoid 3′-hydroxylase (F3′H) and flavonoid 3′,5′-hydroxylase (F3′5′H), which is a key reaction in the metabolism of flavonoids. The third stage is the formation of various anthocyanidins by dihydroflavonols catalyzed by dihydroflavonol 4-reductase (DFR) and anthocyanidin synthase (ANS). The synthesized anthocyanidins are then modified through a series of glycosylation and methylation steps to form stable anthocyanins catalyzed by UDP-glucose: flavonoid glucosyltransferase (UFGT) and methyl transferase (MT).

*CHS* encodes the first key enzyme gene in anthocyanin biosynthesis in plants, which combines one molecule of coumarate-CoA and three molecules of malonyl CoA to form chalcone. Molecular evolution analysis of *CHS* has shown that it is ubiquitous in plants including early land plants and algae of the charophyceae (Schroder, 1997). In ornamental plants, *CHS* has largely been isolated, including petunia (Morgret et al., 2005), phalaenopsis (Han et al., 2006) and herbaceous peony (Zhao et al., 2012b), since it was first reported in parsley by Reimold et al. (1983). And their protein sequences are highly conserved among different plants with ∼80–90% homologies (Beerhues and Wiermann, 1988). *CHS* plays an important role in the synthesis and accumulation of anthocyanins, which induce the results of altering flower color. Transgenic petunia expressing *CHS1* of *Freesia hybrid* shows flower color alteration from white to pink (Sun et al., 2015), and transgenic tobacco expressing *CHS* of *Malus* crabapple displays a higher anthocyanin accumulation and a deeper red petal color compared with control untransformed lines (Tai et al., 2014). In addition, *CHS* expression is often regulated by tissue specificity and different developmental stages, and it has varied sensitivity to environmental stimuli. For example, *CHS* of saﬄower is responsive to wounding, salicylic acid treatment and salinity stress (Dehghan et al., 2014), temperature and UV can induce the expression of *CHS* in *Dryopteris fragrans* (Sun et al., 2014).

*CHI* encodes the second key enzyme gene in plant anthocyanin biosynthesis, which catalyzes the isomerization of chalcone. Chalcone is modified by *CHI* to form flavanone. This product is required in the metabolic branch pathways of flavone, flavonol, proanthocyanidin, and anthocyanin synthesis. Currently, *CHI* has been occurred from Bryophytes through to Angiosperms (Ngaki et al., 2011), and it in plants can be divided into two types according to their catalytic substrates, one type uses 6′-hydroxy chalcone as a substrate, as well as the other can catalyze the isomerization of 6′-hydroxy chalcone and 6′-deoxy chalcone (Chmiel et al., 1983). Regardless of type, whether *CHI* is expressed and its expression level affect flavonoid metabolism in plants, thus affecting flower color development. For example, a decrease in *CHI* expression in carnations, asters, cyclamen and tobacco can result in a greater accumulation of chalcone in petals, turning them yellow (Nishihara et al., 2005).

*F3H* encodes the enzyme gene that catalyzes the hydroxylation of flavanones at C3 to form dihydroflavonol. It is considered a key enzyme at the branch point of the flavonoid biosynthetic pathway. The enzyme can independently regulate metabolism, but often collaborates with the upstream *CHS* and *CHI* products to catalyze the formation of downstream products (Owens et al., 2008). It was shown that the expression patterns and levels of the *F3H* were similar in white, red and blue cineraria petals (Hu et al., 2009). Therefore, the gene was not used in color breeding until 2001 when Zuker et al. (2002) reported that the inhibition of *F3H* expression in an *F3′H* and *F3′5′H* null carnation mutant made orange flowers colorless. To date, the gene has been isolated from ornamental plants, including cineraria (Hu et al., 2009), saussurea (Jin et al., 2005), and herbaceous peony (Zhao et al., 2012b).

*DFR* is another gene encoding a key enzyme in the plant anthocyanin biosynthetic pathway that plays an important role in flower color development. *DFR* belongs to the reduced coenzyme II (nicotinamide adenine dinucleotide phosphate, NADPH)-dependent short-chain reductase family and is encoded by single or multiple gene(s). This enzyme can reduce three types of dihydroflavonols, dihydromyricetin flavonoids, dihydroquercetins, and dihydrokaempferols, to their corresponding colorless anthocyanidins with NADPH. These molecules are further modified to various anthocyanins by downstream gene products (Petit et al., 2007). Because the differences in *DFR* expression and its substrate specificity create color variation in flowers, studies of the mechanisms of its regulation of flower color development have become an important research direction. Currently, *DFR* has been reported to exist in ornamental plants, including Asia lily (Nakatsuka et al., 2003), gentian (Nakatsuka et al., 2005), herbaceous peony (Zhao et al., 2012b), and saussurea (Li et al., 2012). In the study of gene function, Zhao et al. (2012b) studied the expression of *DFR* in different herbaceous peony organs and found that it had the highest expression in the petals that accumulated large amounts of anthocyanins. Similar results have been reported in Asian lily (Nakatsuka et al., 2003) and gentian (Nakatsuka et al., 2005), suggesting that *DFR* may regulate flower color development at the transcriptional level.

*ANS* encodes one of the key enzyme genes in the late stage of anthocyanin biosynthesis. This gene catalyzes the conversion of leucoanthocyanidin to colored anthocyanidin using Fe^2+^ and 2-oxoglutarate (Heller et al., 1985). Studies have shown that *ANS* is encoded by a small gene family in many plants, and these genes have been cloned from ornamental plants, including *Forsythia supensa* (Rosati et al., 1999), gerbera (Wellmann et al., 2006) and herbaceous peony (Zhao et al., 2012b). Rosati et al. (1999) studied the *ANS* gene expression pattern in *Forsythia supensa* and found that null expression of the *ANS* gene resulted in little accumulation of anthocyanins in petals; similarly, the absence of the *ANS* gene sequence was the underlying reason for the color change of lisianthus flowers (Shimizu et al., 2011), suggesting its importance in the regulation of plant colors.

In addition to the structural genes in the anthocyanin biosynthetic pathway, transcription factors also play important roles in flower color development through regulating the temporal and spatial expression of structural genes (Xie et al., 2006). Transcription regulatory genes, also known as transcription factors, are DNA-binding proteins located in the nucleus. They can bind to *cis*-acting elements in promoter regions and regulate the expression of target genes. Currently, there are three main types of transcription factors that affect flower color, MYB, bHLH and WD40 (Ramsay and Glover, 2005). These transcription factors activate or suppress the transcription and expression of target genes through binding to specific DNA sequences and affect protein–protein interactions. Therefore, they regulate anthocyanin synthesis (Zhang et al., 2003). Among the three types of transcription factors that regulate the synthesis of plant anthocyanins, MYB transcription factors have been the most intensively studied. MYB genes have been widely found to regulate the synergistic expression of the structural genes in the plant anthocyanin synthetic pathway at the transcriptional level (Allan et al., 2008; Feng et al., 2010; Pattanalk et al., 2010). Among the three subtypes of MYB transcription factors, R2R3-MYB has commonly been considered closely related to anthocyanin metabolism and regulation (Petroni and Tonelli, 2011; Davies et al., 2012). At present, in-depth studies on this subtype of transcription factor have been reported in vegetables and fruit trees (Kobayashi et al., 2004; Takos et al., 2006; Ballester et al., 2010; Niu et al., 2010). Studies on ornamental plants, in addition to some model plants that were studied in some early reports, such as petunia and snapdragon (Sablowski et al., 1994; Quattrocchio et al., 1999), have been recently reported. For example, in gerbera, *GhMYB10* is closely related to anthocyanin synthesis in leaves, scapes and flowers, and it specifically promotes anthocyanin synthesis in undifferentiated callus tissues and asexual reproductive organs (Roosa et al., 2008). Other examples include the bleaching of gentian flowers due to mutations in *GtMYB3* (Nakatsuka et al., 2008) and the positive regulation of anthocyanin biosynthesis and its effects on organ and tissue-specific anthocyanin accumulation in lily via *LhMYB6* and *LhMYB12* (Yamagishi et al., 2010). Further studies discovered that sequence variations and methylation levels in MYB transcription factor genes also affected anthocyanin accumulation, but this observation was only reported in studies with fruit trees (Espley et al., 2009; Xu et al., 2012) and maize (Das and Messing, 1994; Cocciolone et al., 2001; Robbins et al., 2009).

## Regulation of Flower Color

As one of the major flavonoid pigments, anthocyanins are discussed as above. The flower color development predominantly mediated by anthocyanins can also be regulated by physical and chemical factors and genetic engineering. Therefore, the regulatory factors in flower color development are discussed below.

## Physical Factors

Temperature is a major physical factor which affects flower color. Extreme temperatures will have an impact on flower color development in plants, primarily due to the effect of temperature on anthocyanin accumulation (Lai et al., 2011). In general, high temperatures lead to lighter flower colors due to reduced anthocyanin content in plants such as oriental lily (Lai et al., 2011), rose (Dela et al., 2003), chrysanthemum (Nozaki et al., 2006), and tuberose (Huang et al., 2000). Conversely, low temperatures result in darker flowers because of increased anthocyanin content in plants, such as plantain (Stiles et al., 2007). These phenomena are the result of the suppressed expression of genes involved in anthocyanin biosynthesis, such *CHS*, *F3H* and *DFR*, and thus, the anthocyanin biosynthesis rate is reduced at high temperatures affecting the concentrations of anthocyanins (Lai et al., 2011). In addition, Chen et al. (2000) believed that temperature altered flower color by affecting the cellular structures of petal epidermal cells. At 30∘C, the epidermal cells in petals are arranged in arrays of flat cells, whereas the thickness of the upper epidermis of petals increases at 10–20∘C, which changes the distribution of anthocyanins in these cells, leading to darker petals.

**FIGURE 5:**
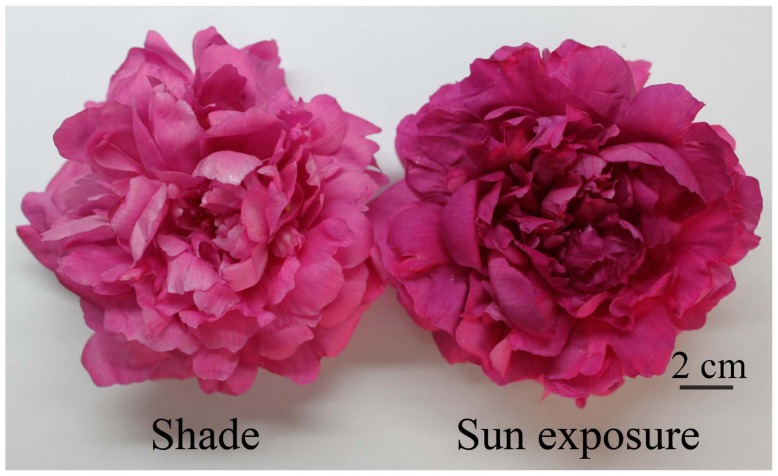
**Herbaceous peony flowers in the bloom stage under sun exposure and shade treatments (Zhao et al., 2012a)**.

Light is another major factor that affects flower color, particularly light intensity, light quality and photoperiod. Based on their requirements for light intensity, plants are classified into heliophytes and sciophytes, and they can only grow well under appropriate light intensities. For example, as a heliophyte, flowers of tuberose are purplish red under strong light intensities, but their color fades under weak light intensities (Huang et al., 2000). This effect also occurs in boronia (KangMo et al., 2007). Shade is a commonly used gardening method for the modification of light exposure. Huang et al. (2000) found that the tuberose flowers cultivated at 25∘C were almost white by 45% shading, but pale reddish-purple under 25 or 0% shade treatment, which was related to the enzyme activity participating the biosynthesis of anthocyanins. Meanwhile, in herbaceous peony, 60% shade caused significantly reduced anthocyanin content and lighter flower color (**Figure 5**), mediated by the synergistic action of structural genes involved in anthocyanin biosynthesis and especially the downregulated expression of *PlPAL*, *PlCHS*, *PlF3H,* and *PlF3′H* (Zhao et al., 2012a).

Additionally, light quality has an impact on flower color. High red light could result in darker flower color of *Hibiscus syriacus* by decreasing Hunter L value but increasing Hunter a value (Young et al., 1997). Moreover, ultraviolet light can also enhance anthocyanin accumulation, UV-B radiation induced an increase in *F3H* enzyme activity of *Reaumuria soongorica* and the accumulation of the products in the flavonoid biosynthetic pathway (total flavonoid and anthocyanin; Liu et al., 2013). And the rich and bright colors of alpine and tropical flowers are all related to the strong ultraviolet light in these regions. In addition, photoperiod also has an impact on flower color. When insolation duration reached more than 12 h, the leaf colors of colored-leaf trees, such as purple-leaf plum, became more vivid and bright (Li and Liu, 1998). The bract color of poinsettia deteriorated when the short-day treatment was ended before bolting (Sun et al., 2006). A prolonged photoperiod led to gradually increased anthocyanin contents in the petals of lisianthus (Uddin et al., 2001).

Water controls the chromaticity of plant organs through its effect on the accumulation of anthocyanins in vacuoles (Zhi et al., 2012). Appropriate water content allows plants to maintain their inherent flower colors for a longer period of time, while water deficiency causes flowers to turn darker (Lai et al., 2011). For example, drought stress induced an increase in *F3H* enzyme activity of *Reaumuria soongorica* and the accumulation of the products in the flavonoid biosynthetic pathway (total flavonoid and anthocyanin; Liu et al., 2013). However, prolonged stress can also cause reductions in anthocyanin content (Li et al., 2009). All these alterations in anthocyanin content led to changes in the colors of plant organs.

**FIGURE 6:**
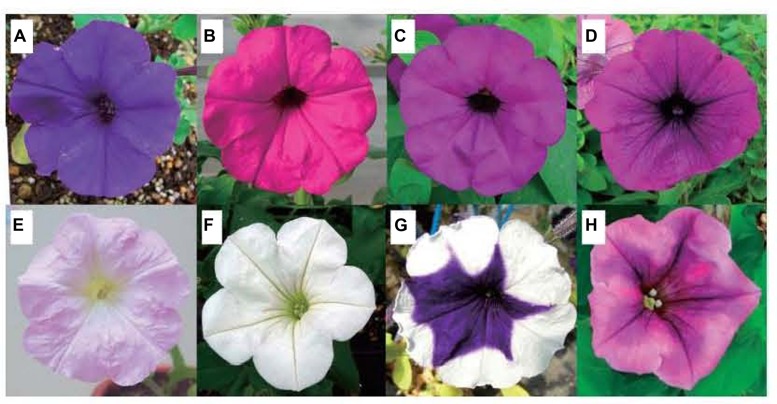
**Parental line of petunia and flower-color mutants by ion beam irradiation (Hase et al., 2010). (A)** Parental line with violet flower color; **(B–H)** Flower-color mutants; **(B)** Magenta; **(C)** purple; **(D)** purple vein; **(E)** light pink; **(F)** white; **(G)** blue picotee; **(H)** burgundy.

In addition to the three physical factors discussed above, pollinators (Adriana et al., 2011), ion beam irradiation (**Figure 6**; Hase et al., 2010; Masayoshi et al., 2012) and gamma rays (Dwivedi et al., 2000; Bala and Singh, 2013) also affect the flower color of ornamental plants.

## Chemical Factors

**FIGURE 7:**
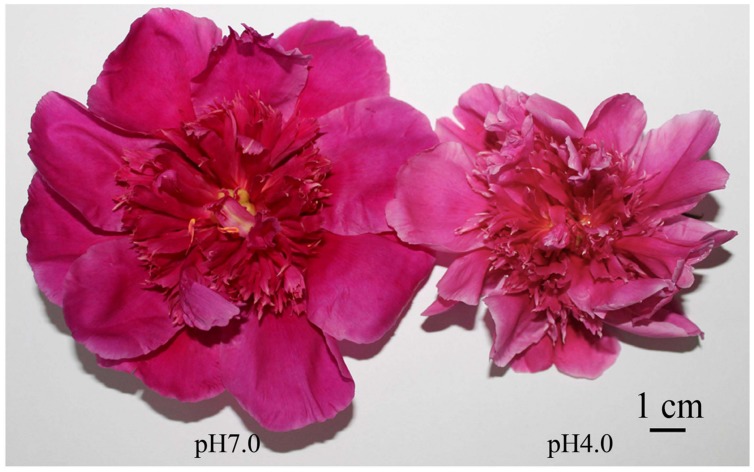
**Herbaceous peony flowers in the blooming stage at pH 7.0 and 4.0 (Zhao et al., 2013)**.

Environmental pH plays an important role in plant color. When *Acer pseudosieboldianum* was planted in acidic soil, autumn leaf coloration occurred early, with a prolonged period of full-color and more splendid leaf color (Han and Gong, 2010). Acidification of the soil was found to affect anthocyanin synthesis in the leaves and enhance leaf color (Sun et al., 2008). Moreover, Liu et al. (2011) found that soil pH did not affect the types of anthocyanins in the petals of lupine. In addition to soil pH, we also examined the effects of pH in irrigation water on flower color (Zhao et al., 2013). When irrigation water pH was at 4.0, herbaceous peony exhibited a lighter flower color (**Figure 7**) with significantly reduced anthocyanin content and markedly increased petal pH. The large decline in the expression level of the anthocyanin biosynthesis structural gene *PlDFR* and the increased expression level of the pH-regulating gene vacuolar Na^+^/H^+^ antiporter1 (*NHX1*) in the petals played vital roles in flower color fading in herbaceous peony.

Mineral nutrients have been widely employed in the regulation of plant color. In the study by Yang et al. (2012), the foliar application of urea, monopotassium phosphate, diammonium phosphate or a combination in climbing rose ‘Angela’ resulted in copious flowers and brighter flower colors. Liu et al. (2009) found that the foliar application of Fe^2+^ improved flower color to different extents. Previous studies reported that *Impatiens hawkerii* exhibited darker flowers under sand culture conditions with 7.41 × 10^-6^ mol/L of aluminum or 3.2 × 10^-7^ mol/L of copper (Li et al., 2005; Li and Fang, 2006), which was due to an increase in soluble sugars and anthocyanins. The effect of the same elements is different in different color varieties. Flower color was significantly improved in the red and orange varieties of lily after the application of a potassium spray, but no effect was observed in yellow lily. The specific mechanism underlying the increased pigment concentrations is still unclear (Burchi et al., 2010).

**FIGURE 8:**
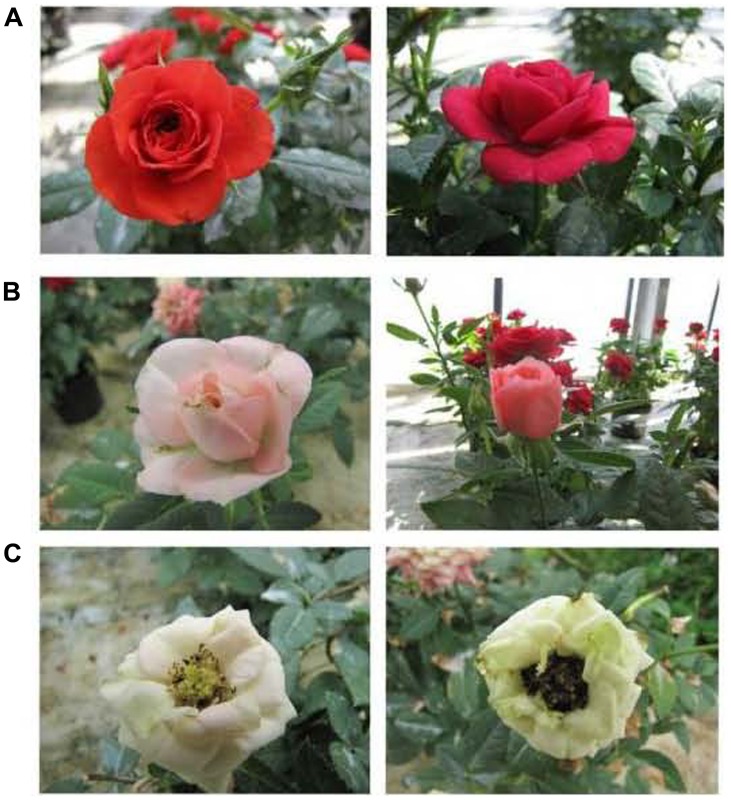
**Flower color changes of two China rose cultivars due to Pro-Ca application (Schmitzer et al., 2012). (A)** Flowers prior to the application of Pro-Ca; **(B)** Flowers 9 days after Pro-Ca application turned light pink; **(C)** Flowers 15 days after Pro-Ca application turned white.

Plant hormones are closely related to flower color, and their effects on color have been examined in a number of studies. Generally, plant growth retardants can effectively improve the color of plants. Currently, this effect has been confirmed for prohexadione-calcium (Pro-Ca; Schmitzer et al., 2012). The petal color of China rose changed from red to light pink and eventually to white after the application of Pro-Ca (**Figure 8**). In addition to anthocyanin content, this phenomenon was found to be directly related to the induction of 3-deoxyflavonoids synthesis (Schmitzer et al., 2012). Furthermore, a study reported that the inhibition of anthocyanidin synthase resulted in red color loss in the ray florets of bronze chrysanthemum after daminozide application which was a well-known chemical inhibitor of the gibberellin biosynthesis (Roepke et al., 2013). In addition, Weiss et al. (1995) found that gibberellin produced in anthers was transported to petals to take effect, where it directly induced the expression of genes, including *CHS*, *CHI*, *DFR* and *UF3GT*.

As a moiety in anthocyanins, carbohydrates provide a precursor substance and energy for anthocyanin synthesis. Therefore, their content directly affects the accumulation of anthocyanins. At present, sucrose, glucose and fructose have been demonstrated to be the main carbohydrates that are effective in promoting anthocyanin accumulation (Neta et al., 2000; Hara et al., 2003; Solfanelli et al., 2006; Zheng et al., 2009; Zhang et al., 2015). In addition, carbohydrates can serve as signaling molecules in the regulation of anthocyanin synthesis-related gene expression and the induction of anthocyanin synthesis via specific signal transduction pathways. A study by Zhang et al. (2015) showed that glucose treatment was found to greatly enhance anthocyanin content and induced the expression of *WD40-2*, *MYB2*, *CHS1*, *CHI1* and *F3′H1* through glucose signaling in tree peony. Neta et al. (2000) found that carbohydrates also regulated anthocyanin synthesis and the expression of genes encoding related enzymes in the corollas of petunia through signaling transduction pathways associated with phosphorylation by hexokinase.

From the perspective of biochemistry and genetics, flower color development is an extremely complex process. Thus, breeding for varieties of different flower colors seems to be outside the scope of traditional breeding techniques. The blooming genetic engineering field brings new ideas and approaches to basic research and variety breeding for flower color in ornamental plants. The identification and characterization of genes encoding key enzymes involved in plant anthocyanin biosynthesis and other genes that influence petal color makes the regulation of plant flower color possible through genetic engineering.

**FIGURE 9:**
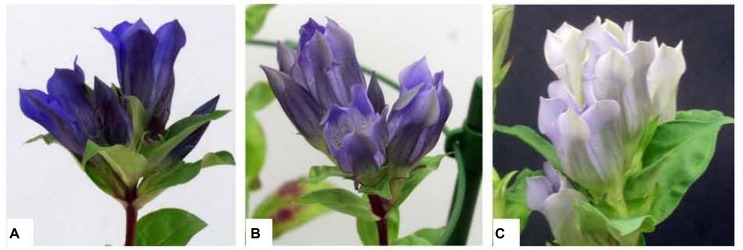
**Flower phenotypes of transgenic gentian plants (Takashi et al., 2010). (A)** The typical flowers of wild-type gentian; **(B)** *5/3′AT*-suppressed transgenic gentian; **(C)** *5/3′AT* and *F3′5′H* double-suppressed transgenic gentian.

Currently, there are two main strategies for the regulation of flower color through transgenic methods. One strategy is to regulate the intrinsic pigment composition and content in petals, while the other is to introduce new pigments into petals. The effects of these two strategies have been confirmed in recent studies (Nishihara and Nakatsuka, 2011). Boase et al. (2010) suppressed the *F3′5′H* gene in cyclamen via antisense inhibition, which led to reduced delphinidin content and elevated cyanidin content, resulting in the petal color changing from purple to red to pink. Takashi et al. (2010) regulated flower color in blue gentian using RNA interference technology. When the anthocyanin 5,3*′*-aromatic acyltransferase gene (*5/3′AT*) was inhibited, the petals became lilac. However, when *5/3′AT* and *F3′5′H* were co-suppressed, the petals were pale blue (**Figure 9**). Meanwhile, the anthocyanin of the petals contents were changed in all transgenic plants. Katsumoto et al. (2007) generated transgenic roses by introducing *F3′5′H* from violet and *DFR* from iris into rose for overexpression. The resulting flowers showed the accumulation of a large amount of delphinidin and a novel blue color in the petals. Zhou et al. (2014) overexpressed *CHI1* from tree peony in tobacco, and the transgenic tobacco petals produced up to three-fold the flavonols and flavones compared to the wild-type. They showed a remarkable reduction in anthocyanin content and flower color intensity.

In addition, Momonoi et al. (2009) identified the vacuole ion transporter *Vit1* in tulips, which made petal cells blue through regulating the accumulation of ions. Verweij et al. (2008) discovered that the *PH5* gene of the petunia generated the blue color by reducing the acidification in vacuoles. In addition, the MYB transcription factor affects flower color through regulating petal cell morphology (Noda et al., 1994; Baumann et al., 2007; Di et al., 2009). All these genes can be used to improve flower color via genetic engineering.

## Concluding Remarks

Flower color in ornamental plants is the result of the joint actions of many factors. To date, a certain understanding of the mechanisms underlying flower color development have been achieved, with in-depth studies on the anthocyanin components, contents, biosynthetic pathways and key genes. In addition, a basic understanding of the types of anthocyanins and their biosynthetic pathways in different ornamental plants has been reached, the regulatory of physical and chemical factors has been explored and their regulative mechanisms are clarified preliminarily. Along with the deepening of research on the functional genomics, proteomics, metabolomics and epigenetics in model plants and the rapid development of high throughput sequencing technology, new opportunities and challenges are brought for researches on the development and regulation of flower color in ornamental plants. And some difficult questions could be solved by drawing on research results in model plants as well as making full use of high-throughput sequencing technology. For example, the complete regulatory mechanisms of flower colors affecting by physical and chemical factors, the interactions among the regulatory factors that can be used for the regulation of flower color and the mechanisms of rare flower color development and directed breeding. However, the huge amounts of data produced in the researches of the development and regulation of flower color in ornamental plants by high-throughput sequencing technology also poses a challenge for our analysis. In order to make great progress in the researches of the development and regulation of flower color in ornamental plants, we must be skilled in bioinformatics, as well as need the infiltration and mergence of multiple subjects.

## Conflict of Interest Statement

The authors declare that the research was conducted in the absence of any commercial or financial relationships that could be construed as a potential conflict of interest.
